# Effect of Different Arbuscular Mycorrhizal Fungi on Growth and Physiology of Maize at Ambient and Low Temperature Regimes

**DOI:** 10.1155/2014/956141

**Published:** 2014-05-05

**Authors:** Xiaoying Chen, Fengbin Song, Fulai Liu, Chunjie Tian, Shengqun Liu, Hongwen Xu, Xiancan Zhu

**Affiliations:** ^1^Northeast Institute of Geography and Agroecology, Chinese Academy of Sciences, Changchun 130102, China; ^2^University of Chinese Academy of Sciences, Beijing 100049, China; ^3^Department of Plant and Environmental Sciences, Faculty of Science, University of Copenhagen, 2630 Taastrup, Denmark; ^4^School of Urban and Environmental Science, Huaiyin Normal University, Huai'an 223300, China

## Abstract

The effect of four different arbuscular mycorrhizal fungi (AMF) on the growth and lipid peroxidation, soluble sugar, proline contents, and antioxidant enzymes activities of *Zea mays* L. was studied in pot culture subjected to two temperature regimes. Maize plants were grown in pots filled with a mixture of sandy and black soil for 5 weeks, and then half of the plants were exposed to low temperature for 1 week while the rest of the plants were grown under ambient temperature and severed as control. Different AMF resulted in different root colonization and low temperature significantly decreased AM colonization. Low temperature remarkably decreased plant height and total dry weight but increased root dry weight and root-shoot ratio. The AM plants had higher proline content compared with the non-AM plants. The maize plants inoculated with *Glomus etunicatum* and *G. intraradices* had higher malondialdehyde and soluble sugar contents under low temperature condition. The activities of catalase (CAT) and peroxidase of AM inoculated maize were higher than those of non-AM ones. Low temperature noticeably decreased the activities of CAT. The results suggest that low temperature adversely affects maize physiology and AM symbiosis can improve maize seedlings tolerance to low temperature stress.

## 1. Introduction

Low temperature is one of the important abiotic factors limiting agricultural productivity and geographical distribution of plants in the world [1, 2]. Maize (*Zea mays* L.) originates from the subtropical regions and is known to be sensitive to low temperature stress. Low temperature adversely affects seed germination and overall growth and productivity of maize plants [3, 4]. There is no doubt that plant membrane is the primary site of low temperature injury which causes the changes of cell membrane structure, lipid composition, and series of metabolic reactions. The membrane damage is accompanied by the increased leakage of electrolytes, production of reactive oxygen species (ROS), and lipid peroxidation [2, 5]. The overproduction of ROS causes the production and scavenging system to be out of balance which causes damage to lipid, protein, DNA, and other important macromolecules [6]. To eliminate extra ROS, plants have evolved specific defense strategy including nonenzymatic and enzymatic antioxidant mechanisms [7]. Furthermore, some osmotic adjustments such as proline and soluble sugar accumulation may protect cellular membrane against low temperature stress.

It is well documented that over 80% of all land plant species form ancient mutualistic interactions with arbuscular mycorrhizal fungi (AMF), soil inhabitants of the monophyletic phylum Glomeromycota [8], which are considered to be obligate biotrophs and complete their life cycle by obtaining the carbohydrate from the host [9, 10]. AMF play key roles in increasing plant growth, nutrient uptake, and ecosystem functioning of sustainable agriculture, as well as enhancing plant tolerance to abiotic stress [10, 11].

At present, there is very little research on the effect of AM symbiosis on physiological process of plant under low temperature conditions. Although some studies have reported AMF's influence on the nutrients uptake and transfer [12, 13], water status [14, 15], and photosynthesis [16, 17] of plants when exposed to low temperature stress, the effect of AMF on lipid peroxidation, osmotic adjustment, and antioxidants of plants at low temperature regime received less attention. Thus, the biomass, contents of MDA, proline and soluble sugar, and activities of antioxidant enzymes of maize plants inoculated with* Acaulospora scrobiculata*,* Glomus etunicatum*,* G. intraradices*, and* G. tortuosum,* respectively, at ambient and low temperature conditions were investigated.

## 2. Materials and Methods

### 2.1. Plant Material and Growth Conditions

Seeds of the maize cultivar Beiyu 288 were surface-sterilized with 75% ethanol for 1 min, then washed 4 times with sterilized distilled water, and thereafter germinated on wet filter paper in Petri dishes at 26°C for 2 days. Three pregerminated seeds were sown in each pot containing 2.4 kg of an autoclaved mixture of black soil and sand. After emergence, the seedlings were thinned to two seedlings per pot. The black soil used in this experiment was collected from a field in Dehui City, Jilin Province, China. The soil was sieved by passing through a 2 mm mesh and sterilized at 98°C for 4 h each for three consecutive days. The soil had a pH of 6.6, 26.9 g kg^−1^ organic matter, 118.8 mg kg^−1^ available N, 18 mg kg^−1^ available phosphorus, and 111 mg kg^−1^ available potassium.

### 2.2. AM Fungus Inoculum

The AM fungi inocula were provided by the Institute of Plant Nutrition and Resources, Beijing Academy of Agriculture and Forestry Sciences, China. The inoculum consisted of soil, spores, mycelia, and infected sorghum root fragments. The AM strains used were* G. etunicatum*,* G. intraradices, A. scrobiculata, *and* G. tortuosum*. Each pot was inoculated with 20 g inocula for mycorrhizal treatment or 20 g sterilized inocula plus 10 mL mycorrhizal fungi-free filtrate from the inocula suspension as the nonmycorrhizal treatment. Mycorrhizal inocula were placed 4 cm below the maize seeds at sowing time.

### 2.3. Experimental Design

The experiment was arranged in a randomized complete block design with five replicates. Treatments were factorial combinations of two factors: (1) inoculation, with* G. etunicatum*,* G. intraradices*,* Acaulospora scrobiculata*,* G. tortuosum, *and nonmycorrhizal control and (2) temperatures, with ambient (25°C) and low temperature (15°C). Before being exposed to the two temperature treatments, the maize seedlings were grown in a greenhouse at 25–28°C with 14 h day light and 75–90% relative humidity for 5 weeks after emergence. Thereafter the seedlings were exposed to either ambient or low temperature treatment for 1 week by placing them into growth chambers. Each pot was weighed and irrigated with sufficient water to avoid soil water deficits, and the plants were fertilized with 100 mL Hoagland's nutrient solution weekly to prevent nutrient deficiency.

### 2.4. Plant Biomass and AM Colonization Analysis

At the end of the treatments, maize plants were harvested. One plant from each pot was used for determination of shoot height, root-shoot ratio, and fresh and dry weight and the other one for measuring physiological parameters. Root and shoot dry weights were determined after oven-drying at 75°C for 48 h. A fraction of the roots was carefully washed, cut into 1 cm long segments, dipped in 10% KOH at 90°C, and stained with 0.01% acid fuchsin in lactophenol [18]. Mycorrhizal colonization rate in the 50 root segments was measured using the gridline intercept method described by Giovannetti and Mosse [19].

### 2.5. Soluble Sugar Determination

Soluble sugar content of maize leaves was determined by the anthrone method [20] using sucrose as the standard. 0.5 g of fresh leaf samples was homogenized with distilled water, placed in a volumetric flask for 1 h, and filtered with filter paper. The reaction mixture contained 1 mL extract and 5 mL anthrone (100 mg anthrone + 100 mL 72% H_2_SO_4_) and was placed in a boiling water bath for 10 min. The absorbance was measured spectrophotometrically at 620 nm.

### 2.6. Proline Content Determination

Proline content of maize leaves was determined using the method of Zhang and Qu [20]. Pure proline was used as a standard. 0.5 g of fresh leaf samples was extracted with 5 mL 3% sulfosalicylic acid, then placed in a boiling water bath for 10 min, and filtered. 2 mL of extract was added to 6 mL assay media containing 2 mL 2.5% ninhydrin solution and 2 mL 17.5 M acetic acid, incubated for 30 min at 100°C, and then cooled. The coloured product was extracted with 4 mL toluene with shaking. The absorbance of the resultant organic layer was measured spectrophotometrically at 520 nm.

### 2.7. MDA Content Measurement

MDA was measured according to the thiobarbituric acid (TBA) reaction as described by Zhang and Qu [20]. 0.5 g of fresh leaf samples was homogenized with 5% trichloroacetic acid (TCA) and centrifuged at 4,000 ×g for 10 min. 2 mL of extract was added to 2 mL 0.6% TBA placed in a boiling water bath for 10 min, and the absorbance at 532, 600, and 450 nm, namely, *A*_532_, *A*_600_, and *A*_450_, respectively, was determined spectrophotometrically. The MDA concentration was calculated according to the formula: 6.45 × (*A*_532_ − *A*_600_) − 0.56 × *A*_450_.

### 2.8. Activities of Antioxidant Enzymes Assays

Fresh maize leaves were homogenized in 5 mL phosphate buffer (0.1 mol/L, pH 7.8), 1% (w/v) polyvinylpolypyrrolidone, and centrifuged at 10,000 ×g for 20 min at 4°C, and the supernatant was collected for superoxide dismutase (SOD), catalase (CAT), and peroxidase (POD) assays.

SOD activity was measured according to Bai et al. [21] based on the ability of SOD to inhibit the reduction of nitroblue tetrazolium (NBT) by superoxide radicals generated photochemically. The reaction mixture contained 50 mM phosphate buffer, pH 7.8, 14 mM methionine, 75 *μ*M NBT, 0.1 *μ*M EDTA, 4 *μ*M riboflavin, and the required amount of enzyme extract. One unit of SOD was defined as the amount of enzyme required to inhibit the reduction rate of NBT by 50% at 25°C.

CAT activity was measured by the disappearance of H_2_O_2_ [20]. The reaction mixture contained 50 mM phosphate buffer, pH 7.0, and 12.5 mM H_2_O_2_. The reaction was initiated by adding the extract and monitoring the change in absorbance at 240 nm for 3 min.

POD activity was determined using guaiacol oxidation in a reaction mixture containing 50 mM phosphate buffer (pH 6.0), 20.1 mM guaiacol, 12.3 mM H_2_O_2_, and enzyme extract. The increase in absorbance was recorded by the addition of H_2_O_2_ at 470 nm for 3 min [21].

### 2.9. Statistical Analysis

The data were analyzed statistically using Microsoft Excel and SPSS 16.0 software. The effects of AM species and temperature treatment on variables passed the test for normal distribution (Kolmogorov-Smirnov test; *P* > 0.05) and homogeneity of variance (Levène test; *P* > 0.05) and were analyzed using two-way ANOVA. Duncan's multiple range test was used to compare the mean values at *P* < 0.05 level. Percentage values were arcsine transformed before statistical analysis.

## 3. Results

### 3.1. AM Fungal Colonization

**Figure 1:**
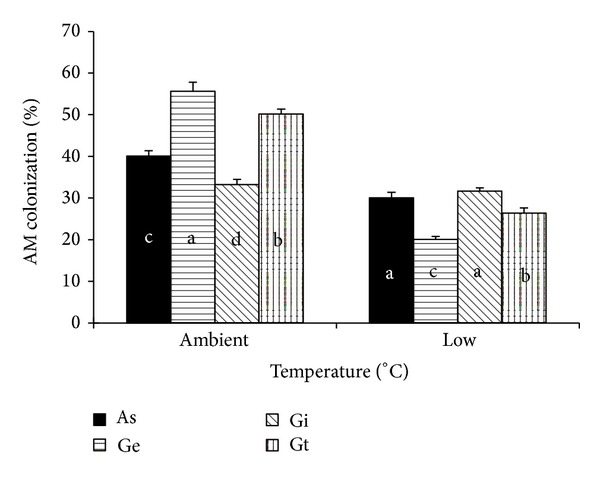
AM colonization of maize seedlings with different AMF strains under ambient temperature and low temperature regimes. As:* Acaulospora scrobiculata*; Ge:* Glomus etunicatum*; Gi:* G. intraradices*; Gt:* G. tortuosum*. Bars represent the mean ± SE of five replicates. For each temperature, different letters on the columns indicate significant difference between the AM strains (*P* < 0.05) by Duncan's test.

There was no AM colonization in the control treatment under both temperature regimes. The results showed that temperature, AMF strain, and their interaction significantly affected the colonization rate (Figure 1). Compared to low temperature treatment, the colonization rates were significantly higher under ambient temperature (ranged from 33.3% to 50.2%). Among the AMF strains,* G. etunicatum* had the highest colonization rate, followed by* G. tortuosum* and* A. scrobiculata*, and* G. intraradices* the lowest. Under low temperature treatment,* G. intraradices* and* A. scrobiculata* had the highest colonization rate, and* G. tortuosum* and* G. etunicatum* the lowest. In relation to the ambient temperature, under low temperature the colonization rate decreased by 17.7% in average across the four AMF strains, and the reduction was particularly evident for* G. etunicatum* (from 55.6% to 20.0%) and* G. tortuosum* (from 50.2% to 26.3%).

### 3.2. Plant Morphology and Growth

Two-way ANOVA indicates that AMF strains had no effect on any of the morphological characteristics of maize seedlings (Table 1). Temperature had significant effects on the height, total dry weight, root dry weight, and root-to-shoot ratio of maize seedlings, while it had no effect on shoot dry weight. Across the five AMF treatments, plant height and total dry weight were significantly higher under ambient than under low temperature, whereas the reverse was the case for the root-to-shoot ratio. In addition, there was no significant interactive effect between temperature and AMF on any of the above variables.

### 3.3. Leaf Soluble Sugar, Proline, and MDA

**Figure 2:**
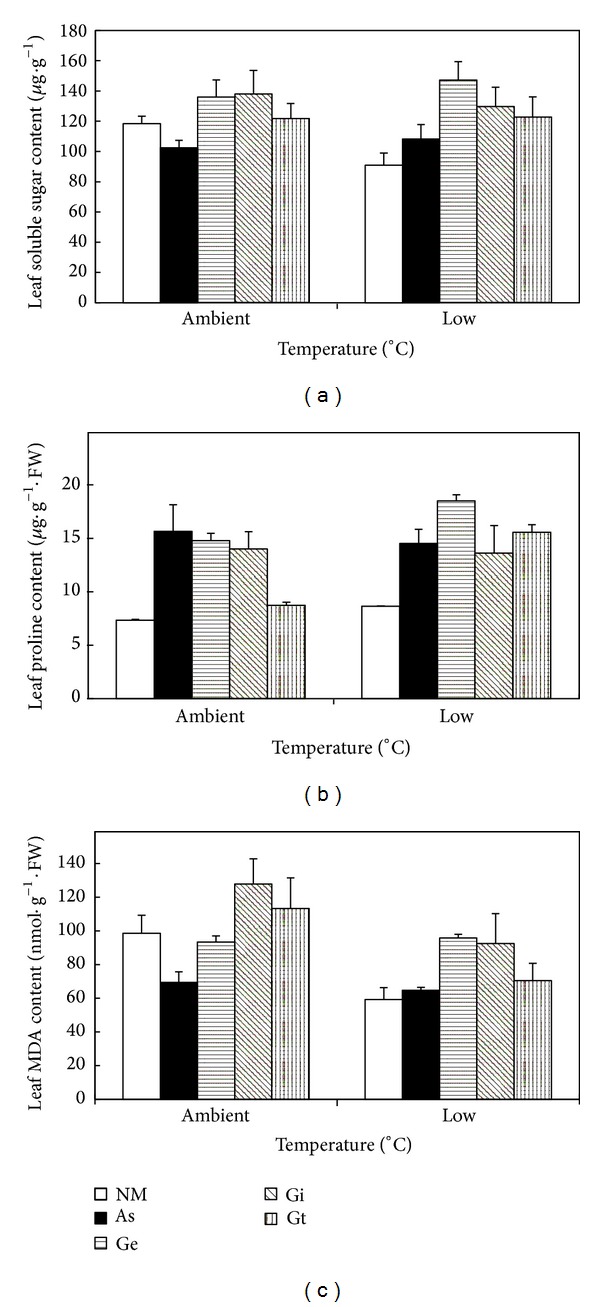
Leaf soluble sugar (a), proline (b), and MDA contents (c) of maize seedlings inoculated with different AMF treatments under ambient temperature and low temperature treatment. NM: non-AM fungus; As:* Acaulospora scrobiculata*; Ge:* Glomus etunicatum*; Gi:* G. intraradices*; Gt:* G. tortuosum*. Bars represent the mean ± SE of five replicates.

Two-way ANOVA indicates that temperature had no effect on leaf soluble sugar content but significantly affected proline and MDA contents (Table 2); AMF treatments significantly affected the contents of leaf soluble sugar, proline, and MDA (Table 2), whereas there were no interactive effects between temperature and AMF on all variables (Table 2). Among the AMF treatments,* G. etunicatum* and* G. intraradices* had the highest leaf soluble sugar content, followed by* G. tortuosum*, and* A. scrobiculata* the lowest which was similar to the non-AMF control plants (Figure 2(a)). Compared to ambient temperature, low temperature significantly increased leaf proline content (Figure 2(b)). Among the AMF treatments,* G. etunicatum* resulted in the highest leaf proline content, followed by* A. scrobiculata*,* G. intraradices,* and* G. tortuosum*; inoculation of any AMF strain significantly increased leaf proline content as compared with the non-AMF plants. In relation to ambient temperature, low temperature significantly decreased MDA content in maize seedlings (Figure 2(c)); across the two temperatures, leaf MDA content was the highest in* G. intraradices*, followed by* A. scrobiculata*,* G. etunicatum*, and* G. tortuosum*, and the lowest in the non-AMF control.

### 3.4. Activities of Antioxidant Enzymes

**Figure 3:**
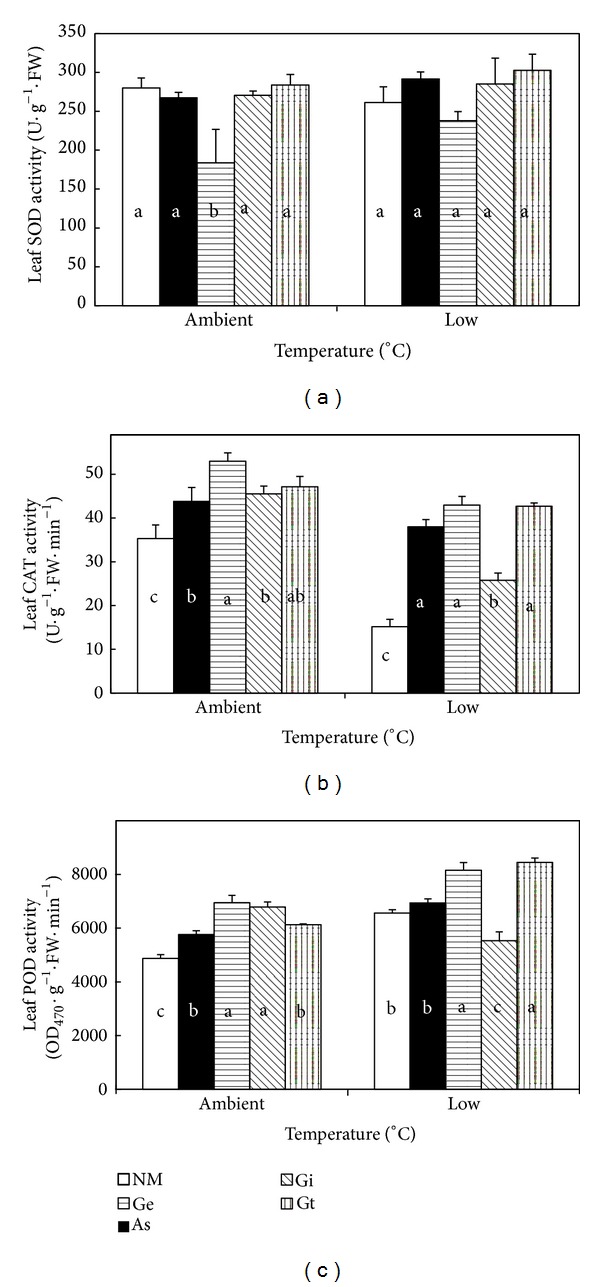
Leaf SOD (a), CAT (b), and POD activities (c) of maize seedlings as influenced by different AMF treatments under ambient temperature and low temperature regimes. NM: non-AM fungus; As:* Acaulospora scrobiculata*; Ge:* Glomus etunicatum*; Gi:* G. intraradices*; Gt:* G. tortuosum*. Bars represent the mean ± SE of five replicates. For each temperature, mean values (±SE) labelled with different letters are significantly different (*P* < 0.05) by Duncan's test.

Two-way ANOVA shows that leaf SOD activity was unaffected by temperature treatments but was significantly influenced by the AMF treatments (Table 2 and Figure 3(a)). Also, there was no significant interactive effect between temperature and AMF treatment on leaf SOD activity. Compared with the non-AMF controls, only* G. etunicatum* showed a significantly lower SOD activity. Temperature, AMF, and their interactions all had significant effects on CAT and POD activities of maize leaves (Table 2 and Figures 3(b) and 3(c)). Across the AMF treatments, CAT activity was significantly lower in low temperature than in ambient temperature. Among the AMF treatments, CAT activity was the highest in* G. etunicatum* and* G. tortuosum*, followed by* A. scrobiculata* and* G. intraradices*, and the lowest for the non-AMF controls. Across the AMF treatments, leaf POD activity was significantly higher under low temperature than those under ambient temperature. Among the two temperature regimes,* G. etunicatum* and* G. tortuosum* had the highest POD activity, followed by* A. scrobiculata* and* G. intraradices*, and the lowest for the non-AMF controls. In addition, different AMF treatments showed different effects on CAT and POD at the two temperature regimes. Under ambient temperature,* A. scrobiculata*,* G. etunicatum*,* G. intraradices,* and* G. tortuosum* increased CAT activity by 24.2%, 50.1%, 29.0%, and 33.6%, respectively, as compared with the respective non-AMF control, while, under low temperature treatment, the increases were 150.2%, 182.8%, 69.9%, and 181.1%, respectively. In relation to the non-AMF control,* A. scrobiculata*,* G. etunicatum*,* G. intraradices,* and* G. tortuosum* enhanced POD activity by 18.1%, 42.6%, 39.2%, and 25.8%, respectively, under ambient temperature. Under low temperature regime,* A. scrobiculata*,* G. etunicatum*, and* G. tortuosum* increased leaf POD activity by 5.8%, 24.3%, and 28.8%, respectively, while* G. intraradices* decreased POD activity by 15.7%.

## 4. Discussion

The AM symbiosis can alter plant growth and physiology under stressful conditions [22]. In the present study, the physiological responses of maize seedlings to low temperature were investigated to explore some of the key elements through which AMF may enhance low temperature tolerance of the plants.

The results showed that low temperature dramatically restrained AM colonization (Figure 1), which was consistent with earlier findings [23, 24]. Moreover, different AMF strains displayed different colonization rates, which suggest that AMF strain has certain selectively to their host plants.

It is generally accepted that both AM symbiosis and temperature affect the growth of plants. In the present study, low temperature significantly affected plant growth in terms of plant height, total dry weight, and root-shoot ratio; however, no positive plant growth responses to AM colonization were observed regardless of temperature treatments (Table 1). Such effect of AMF was also reported in other pot experiments, which could have been due to excess drain of photosynthate to the fungi and failure of AMF to deliver P and other types of nutrition to the plants or short duration of the experiment [25, 26].

Osmotic adjustments play a key role in protecting plant against abiotic stress. The accumulation of small, soluble, and organic molecules, namely, compatible solutes, is a prerequisite for osmotic adjustment [27]. Here it was found that all AM plants had higher soluble sugar content than noninoculated plants under low temperature condition (Figure 2), which may indicate soluble sugar as an osmoprotectant in response to low temperature stress. At ambient temperature, the soluble sugar content of plants with* A. scrobiculata* was lower compared with the non-AM ones likely because of the high amount of sugar allocated to the symbiont in the mycorrhizal plants. Furthermore, proline accumulation increased when maize plants were inoculated with AMF regardless of temperature treatments (Figure 2), similar to earlier findings by Zhu et al. [16]. This implies that AM symbiosis increases the accumulation of proline in maize leaves for osmotic adjustment. Beside contributing to osmotic adjustment, also proline can regulate redox potentials, scavenge hydroxyl radical, protect macromolecules against denaturation, reduce acidity in the cell [28, 29], and thus mitigate the injury of low temperature environment.

It is well known that ROS are continuously produced unintentionally in plants by means of various metabolism reactions and plant cells are well equipped with antioxidants and scavenging enzymes to keep their low under normal growth conditions [7]. However, abiotic stresses such as low temperature stress can increase the rate of ROS production which causes cell damage and destructive oxidative processes such as peroxidation of membrane lipids [30]. Commonly, the level of MDA, generated during lipid peroxidation, is regarded as an indicator of oxidative damage [31]. Recently, Zhu et al. [16] and Latef and Chaoxing [23] reported that low temperature stress increased MDA contents, and AM plants had lower MDA content as compared with the non-AM plants. These suggested that AM symbiosis could alleviate the peroxidation of lipids. Consistent with this, here it was found that both AM symbiosis and low temperature affected the MDA content of maize seedling, which means that AM plants are suffering from varying degrees of oxidative damage under low temperature stress.

Obviously, plants have evolved an elaborate system of enzymatic and nonenzymatic antioxidants which help to scavenge the indigenously produced ROS [32]. Various enzymes involved in ROS scavenging and SOD, CAT, and POD were the key enzymes in the antioxidative defense system. Our study showed that low temperature did not alter the SOD activity and, inoculated with* G. etunicatum,* had lower SOD activity than noninoculated control (Figure 3, Table 2). This does not mean* G. etunicatum* plant was suffering from little or no oxidative stress, but it suggests that it might employ other enzymatic or nonenzymatic routes to eliminate ROS. It was proven that mycorrhizal maize plants had higher CAT and POD activities as compared with the nonmycorrhizal plants regardless of temperature treatments. This result was in agreement with other studies on maize [33] and tomato [23] under low temperature stress. Different AMF had different effect on antioxidative enzyme activities. These findings imply that AM symbiosis could decrease the accumulation of ROS and reduce the damage of oxidative stress by a variety of antioxidant compounds in different ways.

## Figures and Tables

**Table 1:** Effects of AM on plant height, total dry weight, shoot dry weight, root dry weight, and root-shoot ratio of maize seedlings under ambient temperature and low temperature regimes.

Temperature	Inoculation	Plant	Total dry	Shoot dry	Root dry	Root-shoot
		height (cm)	weight (g/plant)	weight (g/plant)	weight (g/plant)	ratio
Ambient	NM	61.1 ± 1.9	0.72 ± 0.07	0.58 ± 0.07	0.14 ± 0.01	0.25 ± 0.03
	As	62.9 ± 1.9	0.75 ± 0.03	0.62 ± 0.03	0.13 ± 0.01	0.21 ± 0.03
	Ge	67.8 ± 2.4	0.72 ± 0.07	0.59 ± 0.07	0.14 ± 0.01	0.25 ± 0.03
	Gi	64.3 ± 1.4	0.77 ± 0.08	0.64 ± 0.07	0.13 ± 0.01	0.21 ± 0.03
	Gt	65.8 ± 2.5	0.92 ± 0.07	0.76 ± 0.06	0.16 ± 0.01	0.20 ± 0.01

Low	NM	57.2 ± 1.2	0.69 ± 0.03	0.55 ± 0.03	0.14 ± 0.01	0.26 ± 0.02
	As	54.9 ± 1.5	0.70 ± 0.15	0.54 ± 0.04	0.15 ± 0.01	0.28 ± 0.02
	Ge	57.3 ± 1.5	0.66 ± 0.05	0.51 ± 0.04	0.15 ± 0.01	0.31 ± 0.03
	Gi	54.7 ± 1.7	0.74 ± 0.08	0.57 ± 0.06	0.18 ± 0.02	0.31 ± 0.02
	Gt	56.7 ± 0.7	0.75 ± 0.05	0.58 ± 0.05	0.17 ± 0.01	0.30 ± 0.03

*P* value						
Temperature (df = 1)		0.001	0.051	0.010	0.010	0.011
AM (df = 4)		0.219	0.077	0.200	0.302	0.936
Temperature × AM (df = 4)		0.395	0.663	0.709	0.540	0.789

NM: non-AM fungus; As: *Acaulospora scrobiculata*; Ge:* Glomus etunicatum*; Gi:* G. intraradices*; Gt:* G. tortuosum*. In each column, values are expressed as mean ± SE.

**Table 2:** Output of two-way ANOVA (*P* value) on the effects of temperature and AMF on physiobiochemical parameters of maize seedlings.

Variables	Soluble sugar	MDA	Proline	SOD	POD	CAT
Temperature (df = 1)	0.611	0.002	0.028	0.170	0.001	0.001
AM (df = 4)	0.008	0.010	0.001	0.005	0.001	0.001
Temperature × AM (df = 4)	0.442	0.156	0.053	0.551	0.001	0.001
